# The role of flavin mononucleotide (FMN) as a potentially clinically relevant biomarker to predict the quality of kidney grafts during hypothermic (oxygenated) machine perfusion

**DOI:** 10.1371/journal.pone.0287713

**Published:** 2023-06-23

**Authors:** Fenna E. M. van de Leemkolk, M. Letizia Lo Faro, Sadr Shaheed, John F. Mulvey, Volkert A. L. Huurman, Ian P. J. Alwayn, Hein Putter, Ina Jochmans, Jan H. N. Lindeman, Rutger J. Ploeg, on behalf of the COMPARE Trial Collaboration and the Consortium for Organ Preservation in Europe (COPE)

**Affiliations:** 1 Department of Surgery, Leiden University Medical Center, Leiden, The Netherlands; 2 LUMC Transplant Center, Leiden University Medical Center, Leiden, The Netherlands; 3 Nuffield Department of Surgical Sciences, University of Oxford, Oxford, United Kingdom; 4 NIHR Oxford Biomedical Research Centre, Oxford University Hospitals Trust, Oxford, United Kingdom; 5 Department of Biomedical Data Sciences, Leiden University Medical Center, Leiden, The Netherlands; 6 Department of Abdominal Transplant Surgery, University Hospitals Leuven, Leuven, Belgium; 7 Transplantation Research Group, Lab of Abdominal Transplantation, Department of Microbiology, Immunology, and Transplantation, KU Leuven, Belgium; Imperial College Healthcare NHS Trust, UNITED KINGDOM

## Abstract

Hypothermic machine perfusion (HMP) provides preservation superior to cold storage and may allow for organ assessment prior to transplantation. Since flavin mononucleotide (FMN) in perfusate has been proposed as a biomarker of organ quality during HMP of donor livers, the aim of this study was to validate FMN as a biomarker for organ quality in the context of HMP preserved kidneys. Perfusate samples (n = 422) from the paired randomised controlled COPE-COMPARE-trial, comparing HMP with oxygenation (HMPO_2_) versus standard HMP in kidneys, were used. Fluorescence intensity (FI) was assessed using fluorescence spectroscopy (excitation 450nm; emission 500-600nm) and validated by fluorospectrophotometer and targeted liquid chromatography mass spectrometry (LC-MS/MS). Fluorescence intensity (FI)_(ex450;em500-600)_ increased over time during machine perfusion in both groups (p<0.0001). This increase was similar for both groups (p = 0.83). No correlation, however, was found between FI_(ex450;em500-600)_ and post-transplant outcomes, including day 5 or 7 serum creatinine (p = 0.11; p = 0.16), immediate graft function (p = 0.91), creatinine clearance and biopsy-proven rejection at one year (p = 0.14; p = 0.59). LC-MS/MS validation experiments of samples detected FMN in only one perfusate sample, whilst the majority of samples with the highest fluorescence (n = 37/38, 97.4%) remained negative. In the context of clinical kidney HMP, fluorescence spectroscopy unfortunately appears to be not specific and probably unsuitable for FMN. This study shows that FMN does not classify as a clinically relevant predictive biomarker of kidney graft function after transplantation.

## Introduction

Hypothermic machine perfusion (HMP) is currently implemented to preserve kidney allografts from deceased donors. It has been demonstrated that HMP reduces the risk of delayed graft function (DGF) and improves graft survival [1–6]. An additional advantage of HMP is that it can facilitate assessment of graft quality prior to transplantation using hydrodynamic parameters and biomarkers [7–10]. Perfusate biomarkers may allow early viability assessment, helping the clinical decision whether to decline or accept the donor kidney, and thus potentially increase the donor pool whilst reducing the number of failed transplants.

Flavin mononucleotide (FMN) is a cofactor for the mitochondrial membrane NADH:ubiquinone oxidoreductase enzyme (complex-I), in addition to a number of other proteins [11]. Whilst FMN is normally tightly bound to complex-I, FMN dissociation from this complex has been reported following ischaemia/ischaemia-reperfusion-induced mitochondrial injury [12–14].

Based on this observation, Muller et al [15]. hypothesised that FMN release in the perfusate might serve as a biomarker of ischaemic injury in liver grafts. Authors reported an association between high FMN release in perfusate and adenosine triphosphate breakdown during oxygenated HMP (HMPO_2_) of deceased donor livers, suggesting that FMN release reflects a compromised energy status of the donor organ. The clinical relevance of this marker during liver perfusion was underpinned by the observation that FMN release during perfusion strongly associates with lactate clearance and coagulation factors at post-transplant days 1 and 2 [15]. In addition, it was found that high perfusate FMN levels correlated with severe allograft dysfunction and early liver graft loss [15].

Similar promising observations for FMN have been reported for porcine kidneys. Darius et al [16]. studied the impact of perfusate oxygen enrichment on transplant outcomes in a porcine kidney autotransplant model. The authors concluded that HMPO_2_ in the first two hours of HMP associated with significantly lower perfusate FMN release compared to standard HMP or end-perfusion HMPO_2_ [16].

These reports characterise perfusate FMN as an easy to asses clinically relevant biomarker that has the potential for real-time monitoring of metabolic integrity and ischaemic-related injury during graft perfusion. The aim of this study was to validate the use of FMN as a predictor of clinical outcomes in the context of HMP (with and without oxygen) of human donor kidneys in kidney transplantation.

## Material and methods

### Clinical samples

**Fig 1:**
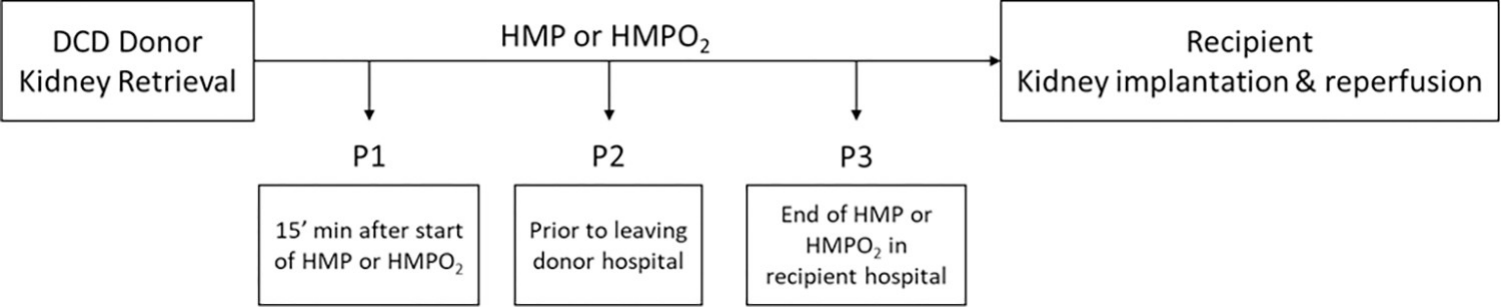
Perfusate sample collection as part of the COPE-COMPARE clinical trial.

Perfusate samples were obtained from the recently published COPE-COMPARE-trial in kidney transplantation carried out by the Consortium for Organ Preservation in Europe (COPE; ISRCTN32967929), Fig 1 [17]. This international multicentre randomised controlled paired trial compared continuous HMPO_2_ with standard HMP in kidneys retrieved from Donation after Circulatory Death donors older than 50 years of age. In total 197 kidney pairs were randomised during this trial. From these kidneys, 197 were assigned to HMPO_2_ and 197 kidneys were assigned to HMP. There was an expected drop-out rate in the trial due to reasons such as no recipient’s written consent, no suitable recipient, combined transplants or the kidney being deemed not transplantable etc. This resulted in 141 transplanted kidneys in the HMPO_2_ arm and 133 transplanted kidneys in the HMP arm. From these transplanted kidneys we included all kidneys (n = 220) where at least one perfusate sample was available (n = 109 kidneys HMPO_2_; n = 111 kidneys HMP). A total of 422 perfusate samples were collected during perfusion as part of the trial protocol and all were used for this analysis (Table 1). The study protocol was approved by the institutional review boards or independent ethics committees in each region of the trial and written informed consent was obtained from the donor’s relatives when required by national law. Written informed consent, including the use of follow-up data stored in a coded way, the collection and storage of biological samples, was obtained from all participants [17]. Samples were stored in a central biobank established by the COPE Consortium for mechanistic studies. The Kidney Assist Transporter (Organ Assist BV, Groningen, the Netherlands) perfusion device was used with University of Wisconsin Machine Preservation Solution (UW-MPS, Bridge to Life) at 4°C and 25mmHg perfusion pressure. Per protocol, perfusates were collected at three different timepoints: 15 minutes after start of HMP or HMPO_2_ (P1); just before leaving the donor hospital (P2); and at the end of HMP or HMPO_2_ (P3) immediately prior to transplantation. P1 and P2 samples were collected by a transplant technician at the donor hospital and stored on ice in a closed box minimizing ambient light exposure. These samples were transported to the transplanting centre together with the corresponding kidney. After P3 collection by another transplant technician in the transplanting centre, all perfusate samples were centrifuged at 1300*g* for 15 minutes. Subsequently, the supernatant was aliquoted, kept on ice in a closed box and transported within six hours to the coordinating centre for storage at -80°C until further analysis.

**Table 1:** Number of perfusate samples collected for different time points as part of the COPE-COMPARE trial.

	HMPO_2_	HMP	Total
*Recipients (n)*	*109*	*111*	*220*
**Start of perfusion (P1) (n)**	59	51	110
**During perfusion (P2) (n)**	53	48	101
**End of perfusion (P3) (n)**	104	107	211

P1 perfusates were obtained 15 minutes after the start of HMP or HMPO_2_; P2 perfusates were obtained just before leaving the donor hospital; P3 perfusates were obtained just before the end of HMP or HMPO_2_.

### FMN analysis in perfusates using fluorescence spectroscopy

After thawing, 150μl of perfusate sample was loaded onto a black clear-bottom non-binding 96-well microplate (Perkin Elmer, Seer Green, United Kingdom (UK)) at room temperature. All available perfusate samples (n = 422) were analysed in duplicate by fluorescence spectroscopy using a microplate reader (CLARIOstar, BMG LABTECH, Aylesbury, UK). The fluorescence was measured in the previously reported FMN-region: excitation 450nm; emission between 500-600nm [15, 18]. A calibration curve was created by serial FMN dilutions (Merck Life Science, Cambridge, UK) in UW-MPS. Correction for the fluorescence intensity (FI)_(ex450;em500-600)_ of a blank sample (UW-MPS) was performed and perfusate samples with FI_(ex450;em500-600)_ below the limit of detection of the assay (i.e. zero or negative FI_(ex450;em500-600)_ following blank correction) were included in the analysis as zero.

### FMN analysis in perfusates using a fluorescence spectrophotometer

Standards were reconstituted in UW-MPS (i.e. 500nM FMN or 500nM Rf), with Rf dissolved initially in acetonitrile to a final volume of ~5%. Clinical perfusate samples included in this analysis were the samples showing the highest (FI)_(ex450;em500-600)_ observed in the previous analysis (n = 13). The full fluorescence emission spectra for these samples were analysed in a FlexStation 3 plate reader (Molecular devices, California, USA) using a bandwidth of 5nm and an excitation wavelength of 440nm (Rf peak excitation is at 442nm, as such an excitation wavelength of 440nm was used in order to measure fluorescence emissions of both compounds). Correction for the blank sample (UW-MPS) was performed, and when below the limit of detection of the assay, included as zero. Standards and clinical perfusate samples were zeroed at 650nm. Data are plotted using a LOWESS smoothing regression curve based on 3 technical replicates (Graph Pad Prism (version 9.0.1)).

### FMN analysis in perfusate using targeted liquid chromatography mass spectrometry

Specificity of the fluorescence measurements was validated using targeted liquid chromatography mass spectrometry (LC-MS/MS) using the Dionex Ultimate 3000 HPLC system coupled with an Orbitrap Fusion mass spectrometer (Thermo Scientific, San Jose, USA). For optimal discrimination the FMN precursor (m/z~457.11) and four dominant fragments (m/z~172.087, 243.088, 359.136, 439.101) were used for identification and quantification of the analyte in standards (S1 Fig) and perfusate samples. Further optimisation and detailed measurement protocols are provided in the S1 and S2 Appendices.

All targeted mass spectrometry data were processed, evaluated, and visualized with Skyline version 21.1.0.2.278 (MacCoss Lab, University of Washington, USA) and Compound Discoverer version 3.1 (ThermoFisher) and subsequently manually verified to ensure correct chromatographic peak selection and integration.

Stability experiments were performed in triplicate on three different conditions to test whether sample processing and storage affected the amount of FMN in perfusate. The three different storage conditions of spiked samples with known FMN concentrations included: (i) storage at 4°C for six hours while kept in the dark; (ii) storage at 4°C for six hours and exposed to ambient light; and (iii) kept overnight at room temperature.

### Endpoints

Primary endpoints for this study were the association between end of perfusion (P3) FI_(ex450;em500-600)_ in the perfusate, and early as well as late post-transplant outcomes. Early post-transplant outcomes included: (i) immediate graft function (IF); (ii) DGF, defined as the need for at least one dialysis treatment in the first week after transplantation; (iii) primary non-function (PNF), defined as a permanent lack of graft-function; and (iv) serum creatinine (SCr) levels at day 5 and day 7 for patients with IF (no dialysis treatment in the first week after transplantation as SCr levels are strongly affected by dialysis treatment). Late post-transplant outcomes (three, six and 12 months) included: (i) estimated creatinine clearance; (ii) graft failure, defined as return to chronic dialysis or pre-emptive re-transplantation, and (iii) biopsy proven rejection. Secondary endpoints explored similar associations for the P1 and P2 timepoints (i.e. beginning and during perfusion, Fig 1) and the delta perfusion (ΔP) measured as the FI_(ex450;em500-600)_ from the P3 perfusates minus the FI_(ex450;em500-600)_ from the P1 perfusates (ΔP = P3 –P1) with early and late post-transplant outcomes.

### Statistical analysis

Continuous data were reported as median with interquartile range. Normality of the data from FI_(ex450;em500-600)_ was approximated by using square root transformation. A linear mixed model, with time (categorical), group (HMP or HMPO_2_) and their interaction as fixed effects and random person effects, was used to compare the two groups (HMP and HMPO_2_) and assess the change of FI_(ex450;em500-600)_ over time. Logistic regression, One-way ANOVA, Pearson correlation, Spearman correlation tests were used to explore associations between FI_(ex450;em500-600)_ and post-transplant outcomes. P-values <0.05 were considered statistically significant (p<0.0045 after Bonferroni correction). Statistical analysis was performed with IBM SPSS Statistics (version 25) and Graph Pad Prism (version 9.0.1).

## Results

A total of 422 perfusate samples collected during perfusion as part of the trial protocol were available for 220 recipients (Table 1) and all were used for this analysis.

### Fluorescence spectroscopy in perfusates

Fluorescence spectroscopy of standard FMN concentrations ranging from 6.2 to 780 nM diluted in perfusion fluid (UW-MPS) showed a linear correlation (*Y* = 253.4×*X*+953.7) (R^2^ = 0.9992; *p<0*.*0001*) (S2 Fig). The limit of blank of the fluorescence spectroscopy assay was 6.7 nM.

**Fig 2:**
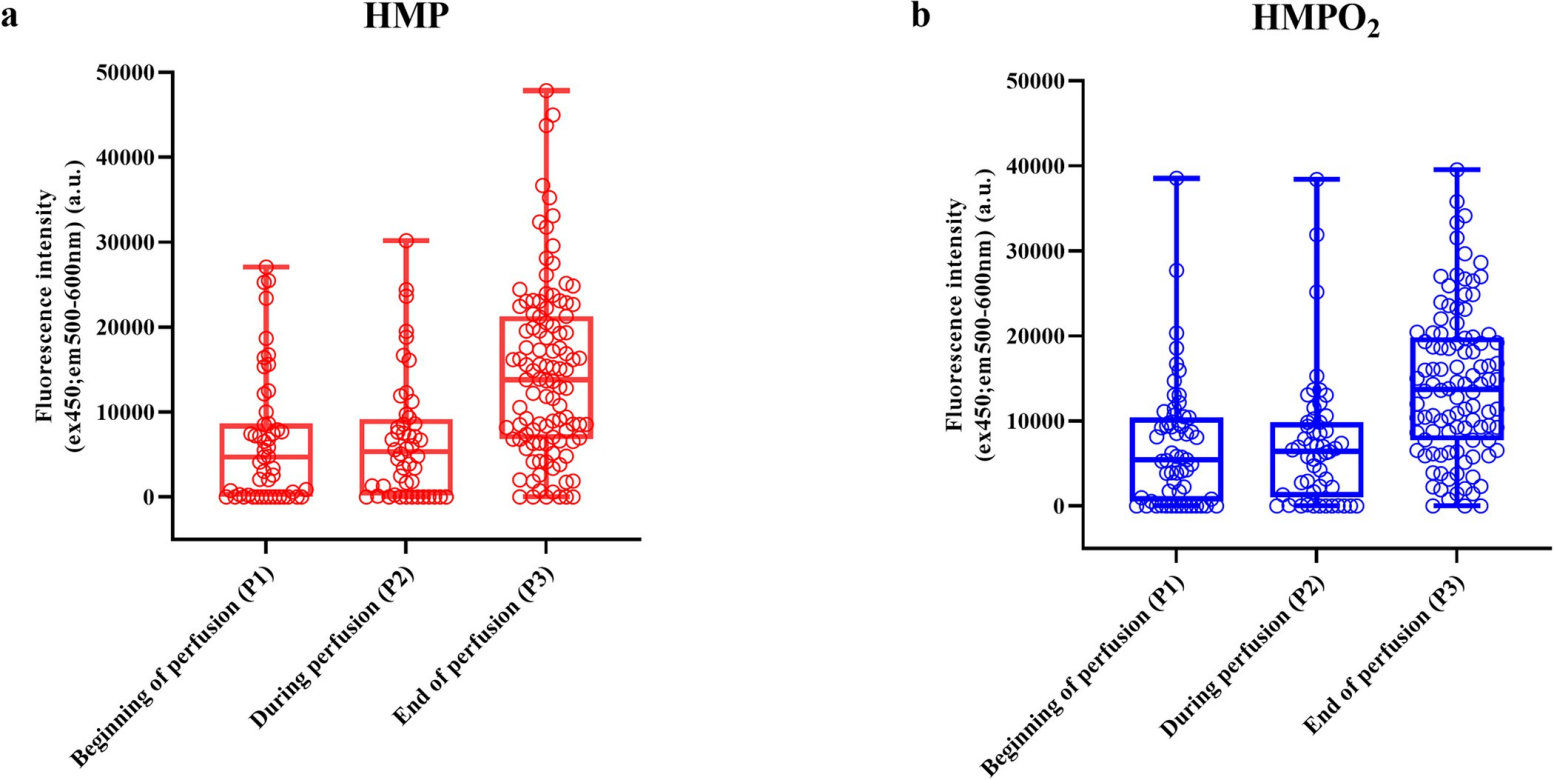
Change in perfusate Fluorescence Intensity (FI)_ex450;em500-600_ over time for HMP (A) and HMPO_2_ (B). P1 perfusates were obtained 15 minutes after the start of HMP or HMPO_2_; P2 perfusates were obtained just before leaving the donor hospital; P3 perfusates were obtained before the end of HMP or HMPO_2_. A linear mixed model was used for analysis and showed a significant increase of FI_ex450;em500-600_ over time during machine perfusion (p<0.0001). This observed increase was similar for both groups (p = 0.83).

Fig 2 illustrates FI_(ex450;em500-600)_ in the perfusates at the different time points of collection (P1, P2 and P3) for both arms of the COPE-COMPARE trial. Linear mixed model analysis indicated a significant increase of FI_(ex450;em500-600)_ over time (*p<0*.*0001*) which was similar for both groups (time * group interaction *p = 0*.*83*). A significant correlation was found between perfusion time (hrs) and FI_(ex450;em500-600)_ for P3 (Spearman r = 0.1920; *p = 0*.*0067*). The median time of perfusion for P2 samples (during perfusion, when the kidney left the donor hospital) and P3 samples (at the end of perfusion) was 4.04 hrs (IQR 2.17–6.26 hrs) and 7.17 hrs (IQR 4.43–9.49 hrs), respectively.

### Fluorescence intensity_(ex450;em500-600)_ and association with post transplantation outcomes

Possible association between FI_(ex450;em500-600)_ measured at the end of perfusion (P3) and clinical post transplant outcomes were evaluated. Since the statistical model indicated similar FI_(ex450;em500-600)_ responses for both groups, the groups were combined to maximise sensitivity.

**Fig 3:**
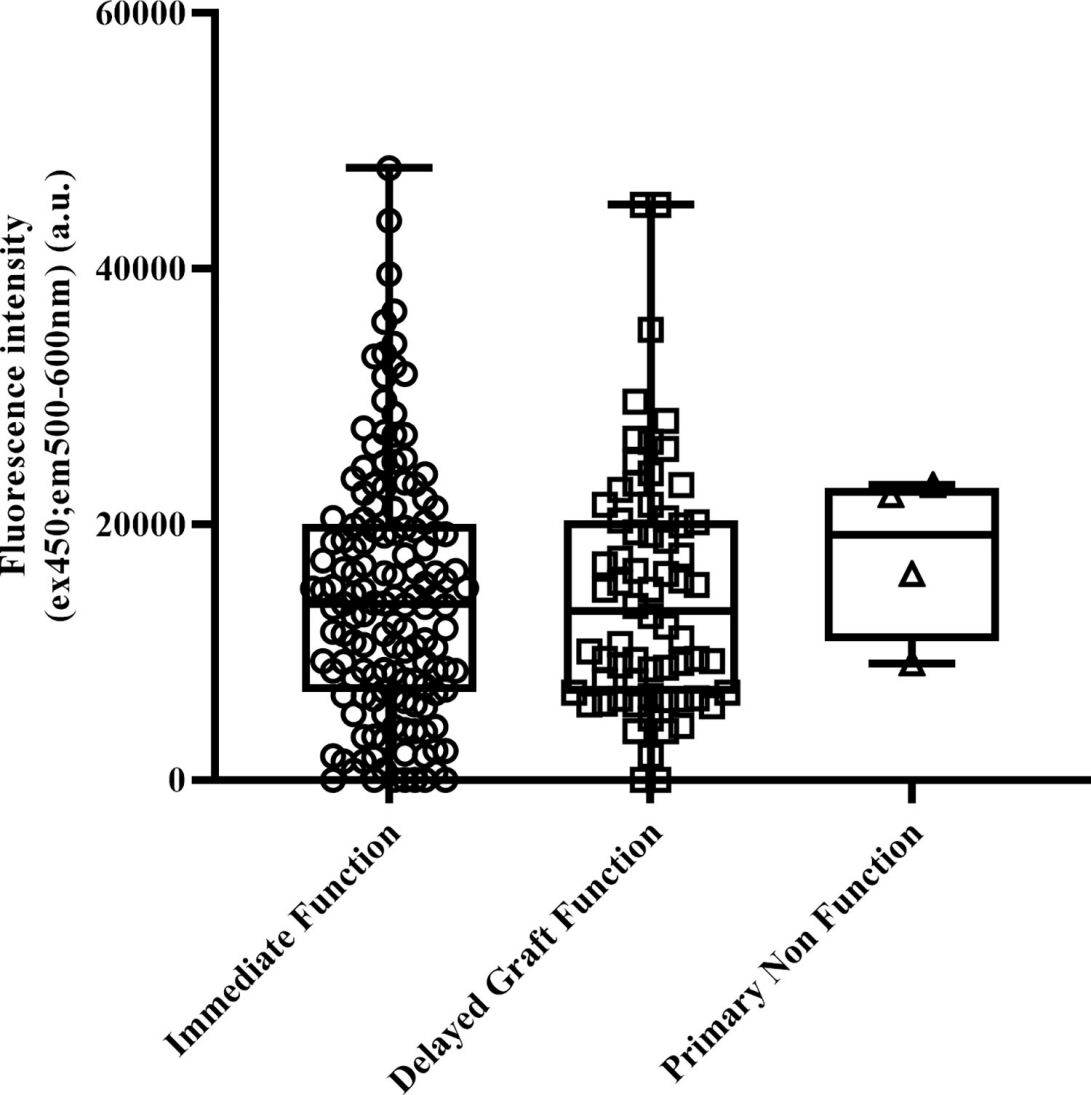
Fluorescence Intensity (FI)_ex450;em500-600_ measured in perfusate samples obtained at the end of perfusion (P3) and correlation with initial graft function of the kidney. No significant differences were observed with one-way ANOVA (p = 0.91) (FI_ex450;em500-600_ data was square root transformed to achieve normality).

No associations were observed between FI_(ex450;em500-600)_ and the primary endpoint early graft function (i.e. IF, DGF and PNF) (Fig 3). The same result was obtained when performing a sensitivity analysis using functional DGF (defined as the absence of a decrease in SCr level by a minimum of 10% per day during three consecutive days in the first week after transplantation), or a stricter definition of DGF that excluded dialysis treatment indicated for fluid overload or hyperkalemia (p = 0.26 and p = 0.59, respectively). No correlation was found between end of perfusion (P3) FI_(ex450;em500-600)_ and day 5 or day 7 SCr (Table 2) in recipients with immediate function. Also, end of perfusion (P3) FI_(ex450;em500-600)_ did not associate with late post-transplant outcomes such as creatinine clearance, graft failure and biopsy proven rejection at 3, 6 and 12 months (Table 3). Similarly, no associations were found between beginning of perfusion (P1), during perfusion (P2) samples or the delta perfusion (ΔP) with secondary endpoints (S1–S4 Tables).

**Table 2:** Correlation of Fluorescence Intensity (FI)_ex450;em500-600_ with early post transplantation outcomes.

	R	p-value
Serum creatinine (μmol/L) Day 5	0.1505	0.11
Serum creatinine (μmol/L) Day 7	0.1352	0.16

Serum creatinine was measured in patients with an immediate functioning graft not requiring dialysis treatment in the first week after transplantation. This was then correlated with Fluorescence Intensity (FI)_ex450;em500-600_ measured at the end of perfusion (P3). Spearman correlation test was used.

**Table 3:** Association of Fluorescence Intensity (FI)_ex450;em500-600_ with post transplantation outcomes.

	3 months		6 months		1 year	
		p-value		p-value		p-value
Creatinine clearance ^†^	-0.078	0.31	-0.003	0.97	-0.118	0.14
Graft failure ^‡^	0.996 [0.98–1.01]	0.63	0.997 [0.98–1.01]	0.75	0.998 [0.98–1.01]	0.81
Rejection ^‡^	0.999 [0.99–1.00]	0.89	0.99 [0.98–1.00]	0.12	1.00 [0.99–1.03]	0.59

^†^ Pearson correlation tests were used for correlation between creatinine clearance and Fluorescence_ex450;em500-600_ measured in perfusates taken at the end of perfusion (P3).

^‡^ Logistic regression analyses were used for association between Fluorescence Intensity (FI)_ex450;em500-600_ and graft failure or rejection.

Data are presented as correlation coefficient (r) or odds ratio with corresponding [95% Confidence Interval].

### Validation of fluorescence spectroscopy of FMN using a fluorescence spectrophotometer

**Fig 4:**
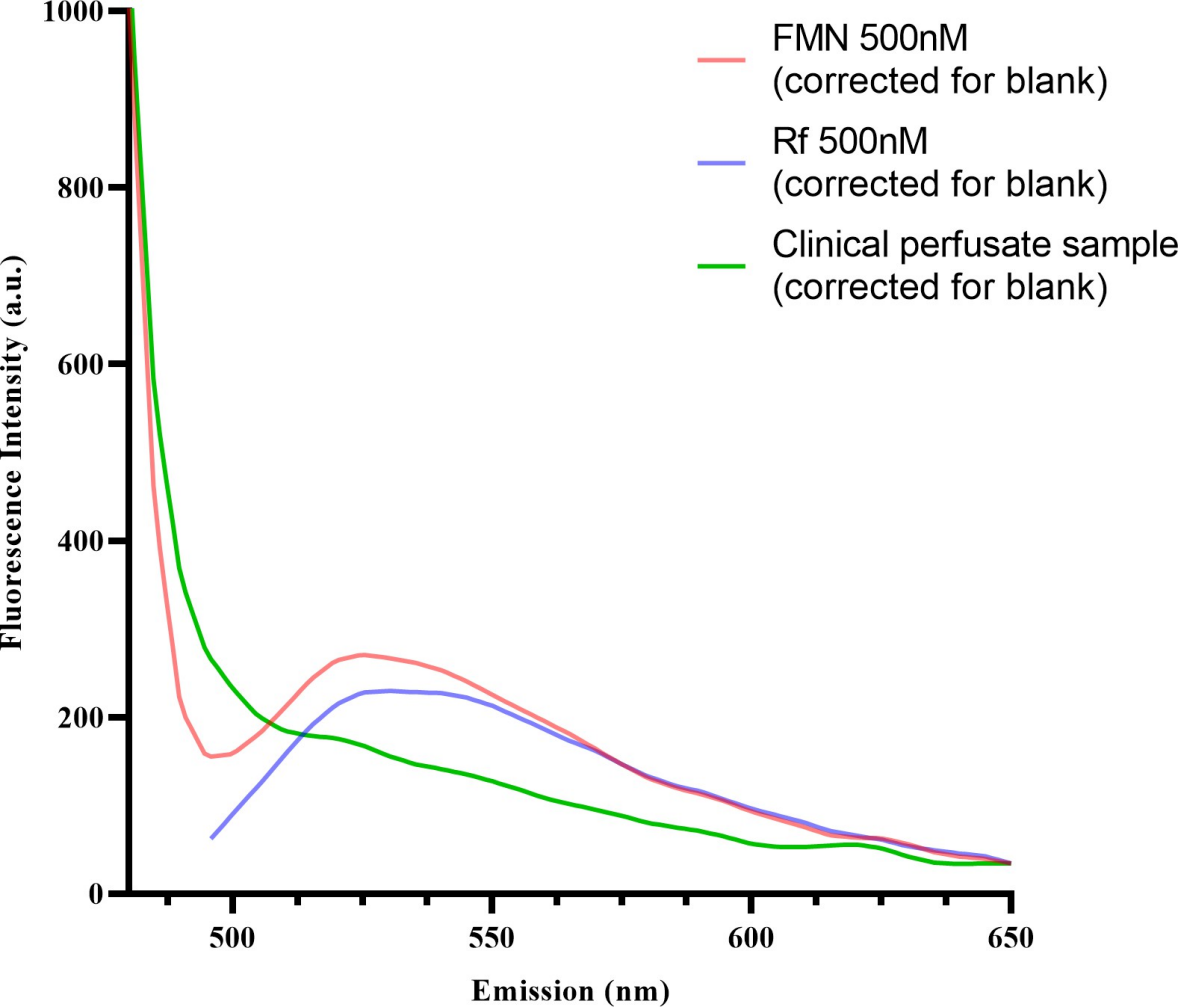
Comparison of fluorescence emission spectra of Flavin Mononuclotide (FMN), Riboflavin (Rf) and clinical perfusate samples. Samples (FMN 500nM, Rf 500nM and clinical perfusate sample (P3) were excited at 440nm and fluorescence emission spectra were recorded (480-650nm). Overlapping emission spectra for standards FMN (500nM) and Rf (500nM) were observed, with an emission peak at approximately 525nm. This emission peak was not observed in the fluorescence emission spectra of the clinical perfusate samples (representative sample P3 is shown), indicating that neither FMN nor Rf are present. Samples were corrected for the blank (containing UW-MPS only) and zeroed at emission 650nm.

The significant increase of fluorescence intensity FI_(ex450;em500-600)_ over time is similarly observed in previous reports, however, the absence of correlation with the fluorescence intensity and post-transplant outcomes in our study differ from previous reports in the literature.[15, 16, 18, 19] To test whether the increase in perfusate FI_(ex450;em500-600)_ was specific for FMN (or the closely related molecule Riboflavin (Rf)), the full fluorescence spectra of perfusate samples were compared with those of standard FMN and Rf. As shown in Fig 4, the overlapping emission peak of FMN and Rf was measured at 525nm. The absence of this characteristic FMN or Rf emission peak in the perfusate samples, suggests that the FI_(ex450;em500-600)_ observed in the initial experiment may not reflect FMN nor Rf.

### Validation of FMN measurement using targeted LC-MS/MS

To rule out any interference due to release of other fluorophores and validate our fluorescence spectroscopy and fluorescence spectrophotometer measurements, we performed the more sensitive technique of targeted LC-MS/MS for FMN (FMN_MS_) analysis. An eight point linear response was obtained with standard concentrations of FMN diluted in perfusion fluid (UW-MPS) (*Y* = 4933.7×*X*+26465) (R2 = 0.9977) (S3 Fig). The limit of detection and limit of quantification of the LC-MS/MS assay were 0.05 picomoles.

**Fig 5:**
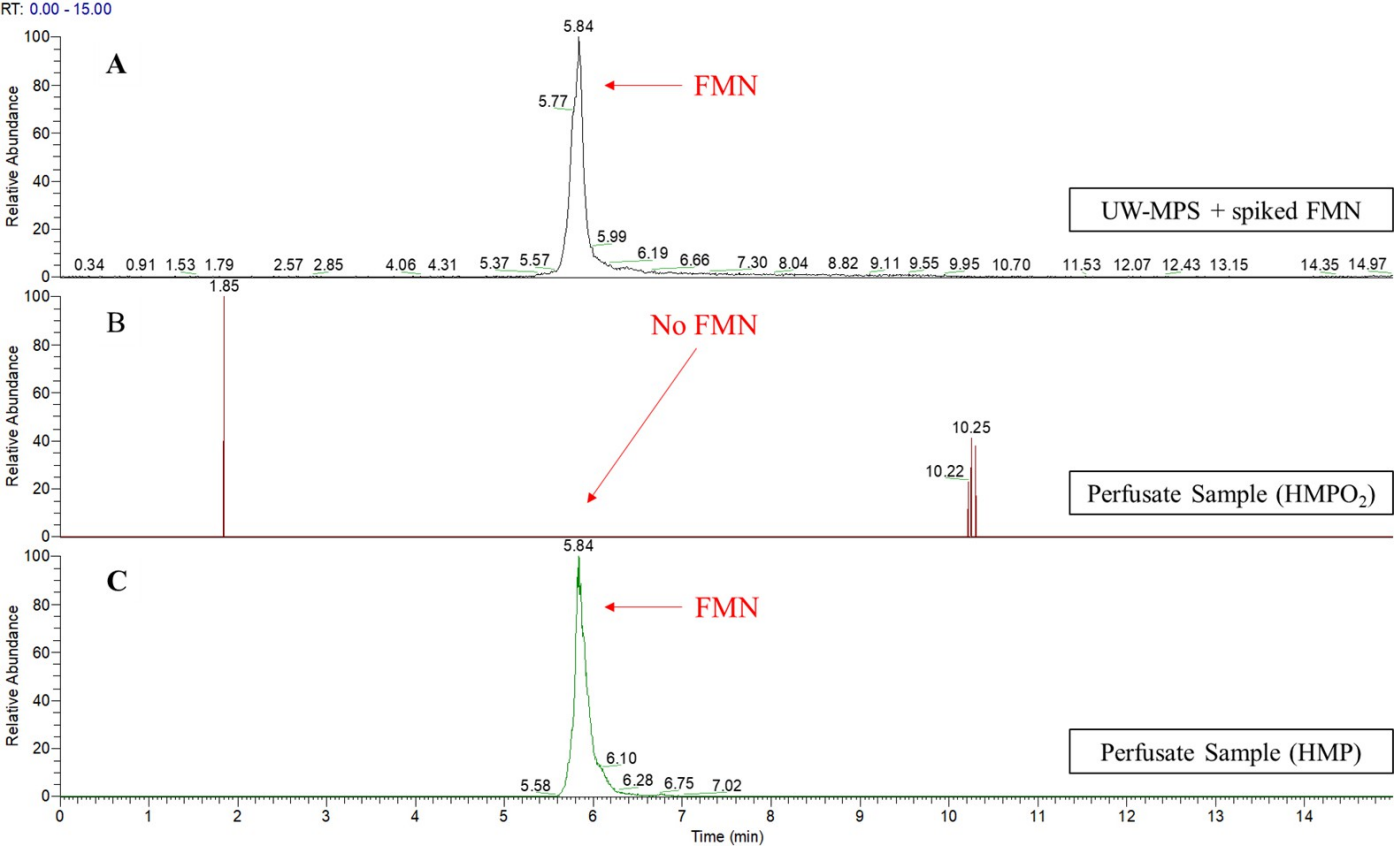
Representative full LC-MS chromatograms of FMN (m/z 457.11) eluted at 5.84 minutes. (A) Chromatogram of a spiked sample with FMN concentration of 1uM. (B) Representative chromatogram of the majority of perfusate samples taken at the end of perfusion (P3) where no FMN was detected. (C) Chromatogram of the only perfusate sample taken at the end of perfusion that was positive for FMN at 5.84 minutes RT.

Validation experiments were performed on a subset of 38 perfusate samples (Fig 5). First, we randomly selected n = 20 samples with n = 10 samples per arm (HMP: P1 n = 2, P2 n = 3, P3 n = 14. HMPO_2_: P1 n = 2, P2 n = 3, P3 n = 14). FMN_MS_ was not detected in these samples. Subsequently, we selected more specifically a subset of n = 18 samples that showed the highest fluorescence intensity (>500nM, when extrapolated from the standard curve of the fluorescence spectroscopy) and FMN was identified in only one sample whilst all other samples with high fluorescence intensity remained negative for FMN. Thus, application of FMN_MS_ identified FMN in only one of the 38 samples (2.6%, 95% CI 0.5–13.5) whilst no FMN_MS_ was detected in the other 37 samples (97.4%, 95% CI 86.5–99.5).

As these observations contrast significantly from work published by other authors, stability experiments were performed to rule out potential artefacts that might have occurred during collection, storage or analysis of our samples. The impact of storage and thawing was tested in samples containing only UW-MPS spiked with standard FMN concentrations (500nM) that were stored at -80°C and subsequently thawed (S4 Fig). This step in sample processing did not affect the stability of FMN_MS_. More importantly, six hours storage on ice (4°C) for samples that were kept in the dark, representing the processing of our samples obtained during the COPE-COMPARE trial, resulted in similar stability of FMN_MS_ (S4 Fig). However, six hours storage on ice (4°C) and exposure to ambient light resulted in 41% reduction of FMN levels as measured by LC-MS/MS (S4 Fig). When stored at room temperature and exposed to ambient light overnight a more pronounced decrease was observed (97% reduction) (S4 Fig). Nevertheless, even when spiked FMN samples were exposed to these extreme environmental conditions, the LC-MS/MS was still able to identify small quantities of FMN, if present.

As our samples were collected per procotol during the COPE-COMPARE trial and subsequently stored for a longer period of time at -80°C, we also investigated whether the absence of FMN measured with LC-MS/MS might relate to prolonged storage. Therefore, clinical kidney perfusate samples (P3) that were stored for a shorter period of time were also analysed. In this subset of nine perfusate samples (HMP n = 5, HMPO_2_ n = 4) samples were stored in -80°C for a median time of 4 months. Again, no FMN was detected by the LC-MS/MS in these samples.

## Discussion

The aim of this study was to confirm and validate the clinical role of FMN as a helpful clinical biomarker during hypothermic machine perfusion of donor kidneys, predicting the quality of the kidney grafts prior to transplantation. Following previous positive reports, fluorescence spectroscopy was used to analyse perfusate samples from donor kidneys that had been included in a randomised controlled multicentre trial comparing oxygenated and non-oxygenated hypothermic machine perfusion (HMP versus HMPO_2_). In this first large clinical study in kidney transplantation to assess the role of FMN as a predictive biomarker, we observed comparable increases of fluorescence intensity (FI)_ex450;em500-600_ over time in both groups, however, no correlation was found with early or late post-transplant outcomes. Subsequent validation experiments using targeted LC-MS/MS identified the presence of FMN in only one out of a validation subset of 38 samples that expressed the highest FI_ex450;em500-600_ values. Therefore, this study suggests that in the context of the cold perfused human kidney, fluorescence has low specificity for FMN detection, and fluorescence in the FMN region may not be usable as a predicting clinical biomarker of kidney transplant outcomes.

Our observations in clinical kidney transplantation are not in line with earlier reports [15, 16, 18, 19]. Darius et al. [16] assessed fluorescence intensity (FI) during the first two hours of HMP in a porcine kidney autotransplant model [16]. Kidneys were randomised to either start-preservation oxygen enrichment (2hrs HMPO_2_ + 20hrs HMP) or end-preservation oxygen enrichment (20hrs HMP + 2hrs HMPO_2_). Although the article refers to two historical pre-clinical control groups using conditions that appear to be comparable to the two arms in our subsequent clinical trial (i.e. 22hrs standard HMP and 22hrs HMPO_2_) unfortunately, we could not find any data for 22hrs HMPO_2_ to directly compare to our findings. An important question is whether the author’s conclusion that superior outcomes correlate with reduced fluorescence intensiy in perfusate is entirely justified. In particular, the statistical approach used is interfered by baseline differences. It is likely that different conclusions might have been reached when using a more appropriate linear mixed model analysis [20].

Wang et al [19]. analysed FI in stored, blood-based perfusates obtained from kidneys during normothermic machine perfusion (NMP) and abdominal normothermic regional perfusion. They found that FI in the NMP setting had a predictive value for post-transplant renal function in terms of initial graft function. NMP, using red blood cell enriched perfusate is obviously a different condition from acellular HMP. However, an important question may be whether direct fluorescence spectroscopy can be helpful in the presence of red blood cells in the perfusion fluid. Both riboflavin and its derivatives FMN and FAD are present in blood during normal physiology with median plasma concentrations of 10.5, 6.6 and 74nmol/L for riboflavin, FMN and FAD, respectively. This raises the point whether any FMN detected in this study actually reflects injury in the kidney or merely represents an innocent bystander due to the presence of third-party blood [21].

To date, several studies have used various excitation and emission fluorescence spectra to measure FMN [12, 14, 15, 18, 19, 22–24]. Although the direct methodology of fluorescence spectroscopy is straightforward and compatible with real-time measurements, its specificity can be interfered by several endogenous fluorophores that fluoresce at the same wavelength (500-600nm) following excitation at 450nm [25]. Similarly, in our clinical study we refer to the fluorescence intensity in the FMN region (500-600nm) as fluorescence intensity (FI)_ex450;em500-600_, as it is not a guarantee that the compound in the perfusate samples responsible for the fluorescent signal is actually FMN.

Support for the increase of non-specific fluorescence during perfusion observed in our study and also in the previously cited studies [16, 19] can be attributed to other interfering factor(s) such as other flavins (e.g. riboflavin (Rf) or FAD), tetra-porphyrin ring catabolites or metabolites with overlapping excitation and emission fluorescence spectra in the FMN region. For instance, tissue FAD content is present in 10-fold excess of FMN [26], yet this interference by other flavins (e.g. Rf) is not supported by our fluorescence spectrophotometer experiments (Fig 4) LC-MS/MS. To rule out non-specific fluorescence and confirm the presence of FMN in kidney perfusate, we used targeted LC-MS/MS technology. Despite excellent sensitivity, FMN_MS_ was only identified in one out of 38 samples in the validation subset. This suggests that the increase in FI_(ex450;em500-600)_ observed in our clinical study might indeed relate to release of other fluorophores with (partial) overlapping fluorescent spectra.

A limitation of our study might be the sample handling during the COPE-COMPARE trial. Sample processing and storage in the COPE-COMPARE trial took place in the transplanting and coordinating centre, respectively. As a consequence, perfusate samples obtained at the beginning and during perfusion (P1 and P2) were temporarly maintained on ice in a closed lid dark box. After collection of the P3 perfusate sample just before the end of perfusion, all samples were jointly centrifuged, the supernatant was placed back on ice in a closed lid box for the duration of transport to the coordinating centre, aliquoted and frozen at -80°C, to be thawed only for analysis.

To ensure that our results were correct and rule out that the lack of confirmation of FMN was not due to an artefact, we performed several sample processing stability experiments. In line with Wang et al., [19] fluorescence of spiked FMN samples was found to remain stable during long-term storage at -80°C. Importantly, using LC-MS/MS for additional perfusate samples (n = 9) that were stored for a shorter period of time at -80°C, samples still remained negative for FMN_MS_. Regarding the spiked FMN samples, we did observe partial degradation of FMN_MS_ as measured by LC-MS/MS when spiked samples were exposed to ambient light and kept at higher temperatures. However, this did not result in full degradation of FMN_MS_, since LC-MS/MS analysis was still able to identify a peak area.

Whilst we investigated the potential value of FMN as a predictor of outcome in clinical kidney transplantation, the use of FMN as a viability tool has primarily been studied in the setting of ex-situ HMP of human [15] and rat [18] livers. Muller et al. [15] performed fluorometric assessment of FMN in machine perfusates during HMPO_2_ of human liver grafts. It was concluded that real-time optical measurement of FMN provides a fast prediction of the liver graft function and therefore might be a highly clinically relevant biomarker in liver transplantation. The authors state that they have validated the measurement of FMN in the perfusate by using targeted LC-MS/MS, unfortunately, the LC-MS/MS results were not included in the manuscript. Possibly different sensitivities between kidney and liver, as well as the response to ischaemic injury of mitochondria, may explain the different results between the liver and this clinical kidney study.

In conclusion, despite a thorough effort to trace FMN in this large perfusate sample study of donor kidneys included in a controlled randomised clinical multicentre trial in kidney transplantation evaluating hypothermic kidney perfusion with and without oxygenation, we could not find any correlation of an increased fluorescent biomarker in the perfusate with outcomes after kidney transplantation nor identiy FMN in the kidney perfusate. The data of this clinical study suggest that FMN in the perfusate unfortunately cannot reliably be used as a clinical biomarker to predict kidney graft function after transplantation.

## Supporting information

S1 Fig Fragmentation of FMN (m/z~457.11) on an Orbitrap Fusion mass spectrometer (ThermoScientific) in positive polarity mode. For optimal discrimination the FMN precursor (m/z~457.11) and four dominant fragments (m/z~172.087, 243.088, 359.136, 439.101) were used for identification and quantification of the analyte in standards and perfusate samples.
(TIF)

S2 Fig Calibration curve fluorescence spectroscopy. A linear correlation was obtained after standard concentrations of FMN (nM) ranging from 6.2 to 780 nM were diluted in perfusion fluid (UW-MPS) (*Y* = 253.4×*X*+953.7) (R^2^ = 0.9992; *p<0*.*0001*).
(TIF)

S3 Fig Calibration curve for targeted liquid chromatography mass spectrometry. An eight point linear response was obtained after standard concentrations of FMN were diluted in perfusion fluid (UW-MPS) (*Y* = 4933.7×*X*+26465) (R2 = 0.9977). The limit of detection and limit of quantification of the LC-MS/MS assay were 0.05 picomoles.
(TIF)

S4 Fig Representative LC-MS/MS chromatograms of FMN (m/z 457.11) during stability experiments. UW-MPS samples spiked with standard FMN concentration (500nM). (A) Storage at -80°C. (B) Storage overnight at room temperature and exposure to ambient light. (C) Storage for six hours on ice (4°C) and exposure to ambient light. (D) Storage for six hours on ice (4°C) and kept in the dark.
(TIF)

S1 Table Correlation of Fluorescence Intensity (FI)_(ex450;em500-600)_ at the beginning of perfusion (P1), during perfusion (P2) and the delta perfusion (ΔP) with early post transplantation outcomes. Serum creatinine was measured in patients with an immediate functioning graft not requiring dialysis treatment in the first week after transplantation. This was then correlated with fluorescence intensity (FI)_(ex450;em500-600)_ measured at the beginning of perfusion (P1), during perfusion (P2) and the delta perfusion (ΔP = P3-P1). Spearman correlation test was used.
(DOCX)

S2 Table Association of Fluorescence Intensity (FI)(ex450;em500-600) at the beginning of perfusion (P1) with post transplantation outcomes. ^†^ Pearson correlation test was used for correlation between creatinine clearance and FI_(ex450;em500-600)_ measured in perfusates taken at the beginning of perfusion (P1). ^‡^ Logistic regression analyses were used for association between FI_(ex450;em500-600)_ and graft failure or rejection. With regards to rejection at one year, the numbers were too small to perform the analysis. Data are presented as correlation coefficient (r) or odds ratio with corresponding [95% Confidence Interval].
(DOCX)

S3 Table Association of Fluorescence Intensity (FI)(ex450;em500-600) during perfusion (P2) with post transplantation outcomes. ^†^ Pearson correlation test was used for correlation between creatinine clearance and FI_(ex450;em500-600)_ measured in perfusates taken during perfusion (P2). ^‡^ Logistic regression analyses were used for association between FI_(ex450;em500-600)_ and graft failure or rejection. With regards to rejection at one year, the numbers were too small to perform the analysis.Data are presented as correlation coefficient (r) or odds ratio with corresponding [95% Confidence Interval].
(DOCX)

S4 Table Association of Fluorescence Intensity (FI)(ex450;em500-600) from the delta perfusion (ΔP) with post transplantation outcomes. ^†^ Spearman correlation test was used for correlation between creatinine clearance and FI_(ex450;em500-600)_ from the delta perfusion (ΔP) measured as (P3) perfusates taken at the end of perfusion–(P1) perfusates taken at the beginning of perfusion.^‡^ Logistic regression analyses were used for association between FI_(ex450;em500-600)_ and graft failure or rejection. With regards to rejection at one year, the numbers were too small to perform the analysis.Data are presented as correlation coefficient (r) or odds ratio with corresponding [95% Confidence Interval].
(DOCX)

S1 Appendix Material & methods. FMN analysis in perfusate using targeted liquid chromatography mass spectrometry.
(DOCX)

S2 Appendix Protocol fluorescence measurements of FMN. (DOCX)

S1 Dataset Graphpad. (SAV)

S2 Dataset SPSS. (PZFX)

S1 File (DOCX)
