# Supplementary figures and images for: The role of flavin mononucleotide (FMN) as a potentially clinically relevant biomarker to predict the quality of kidney grafts during hypothermic (oxygenated) machine perfusion
Source: PLoS One. 2023 Jun 23;18(6):e0287713. doi: 10.1371/journal.pone.0287713 (PMC10289320; doi:10.1371/journal.pone.0287713)

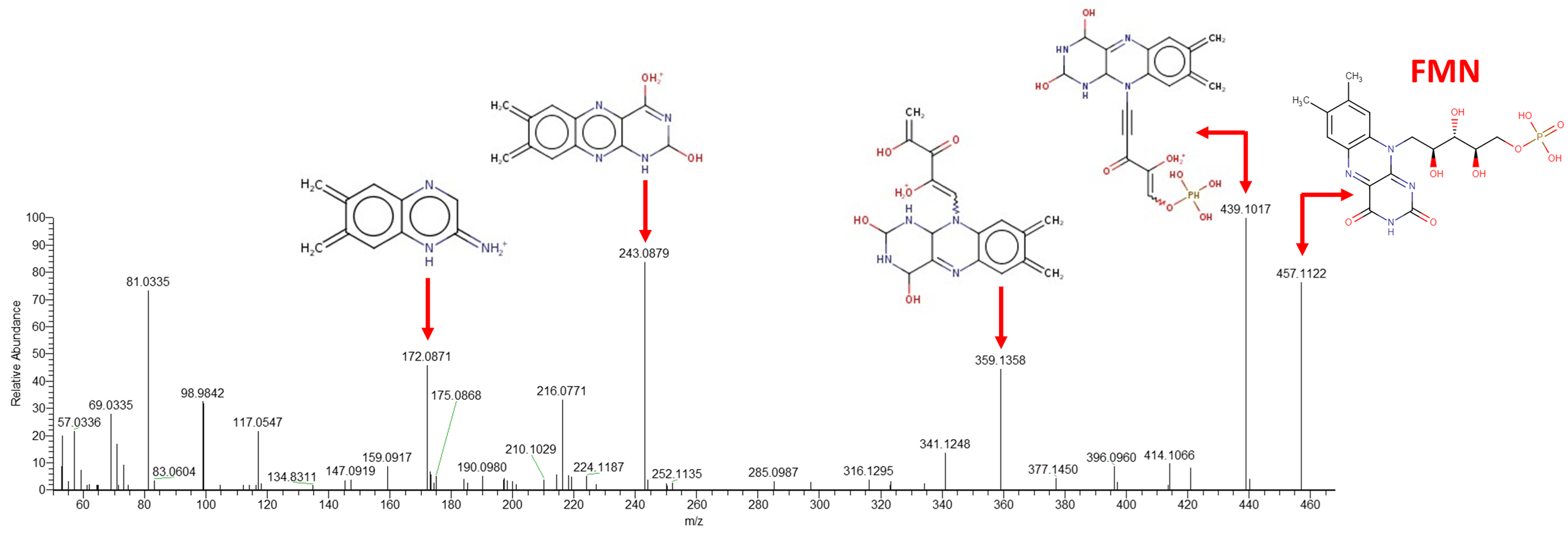

Supplement: S1 Fig — For optimal discrimination the FMN precursor (m/z~457.11) and four dominant fragments (m/z~172.087, 243.088, 359.136, 439.101) were used for identification and quantification of the analyte in standards and perfusate samples. (TIF) [file pone.0287713.s001.tif]

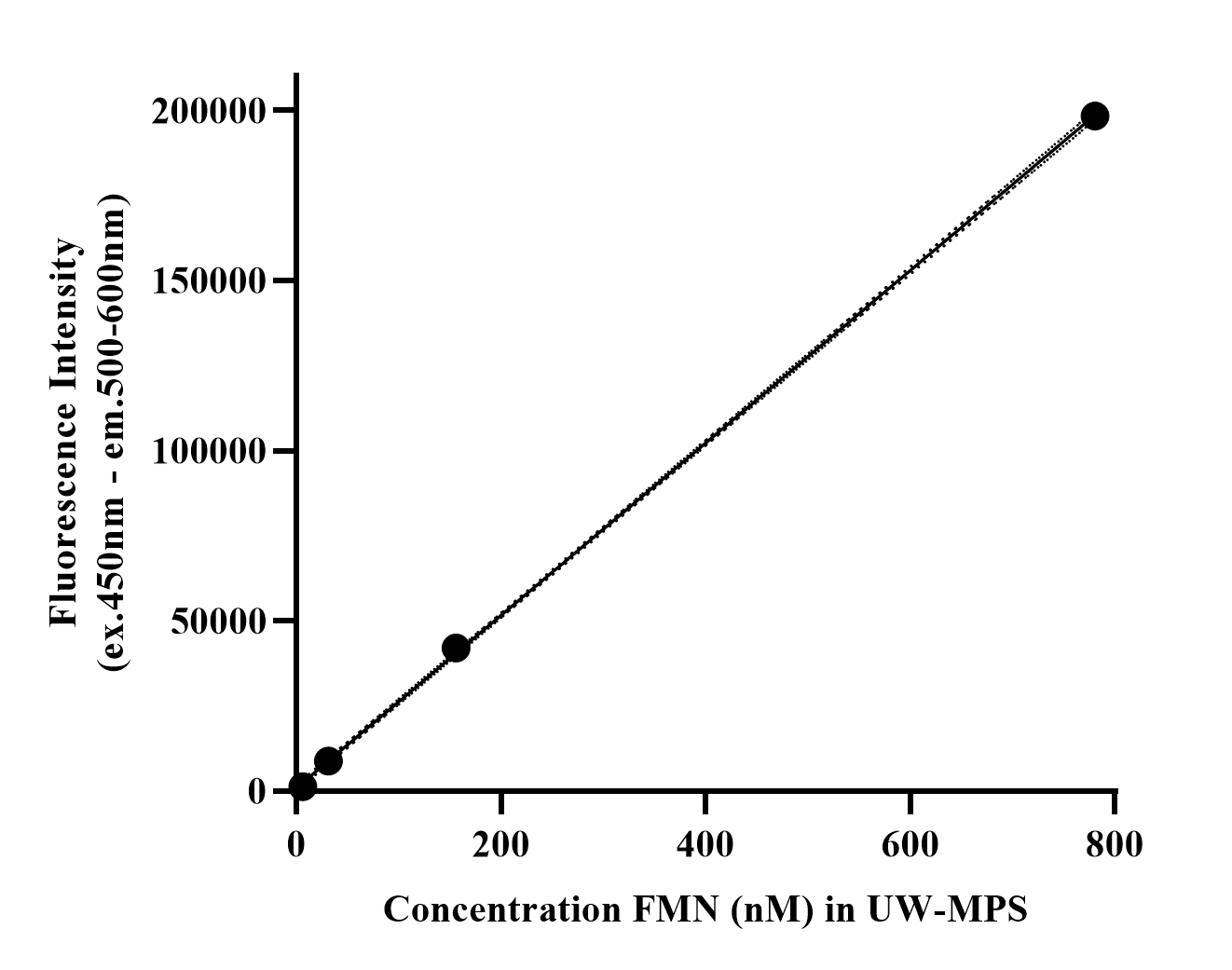

Supplement: S2 Fig — A linear correlation was obtained after standard concentrations of FMN (nM) ranging from 6.2 to 780 nM were diluted in perfusion fluid (UW-MPS) (Y = 253.4×X+953.7) (R2 = 0.9992; p<0.0001). (TIF) [file pone.0287713.s002.tif]

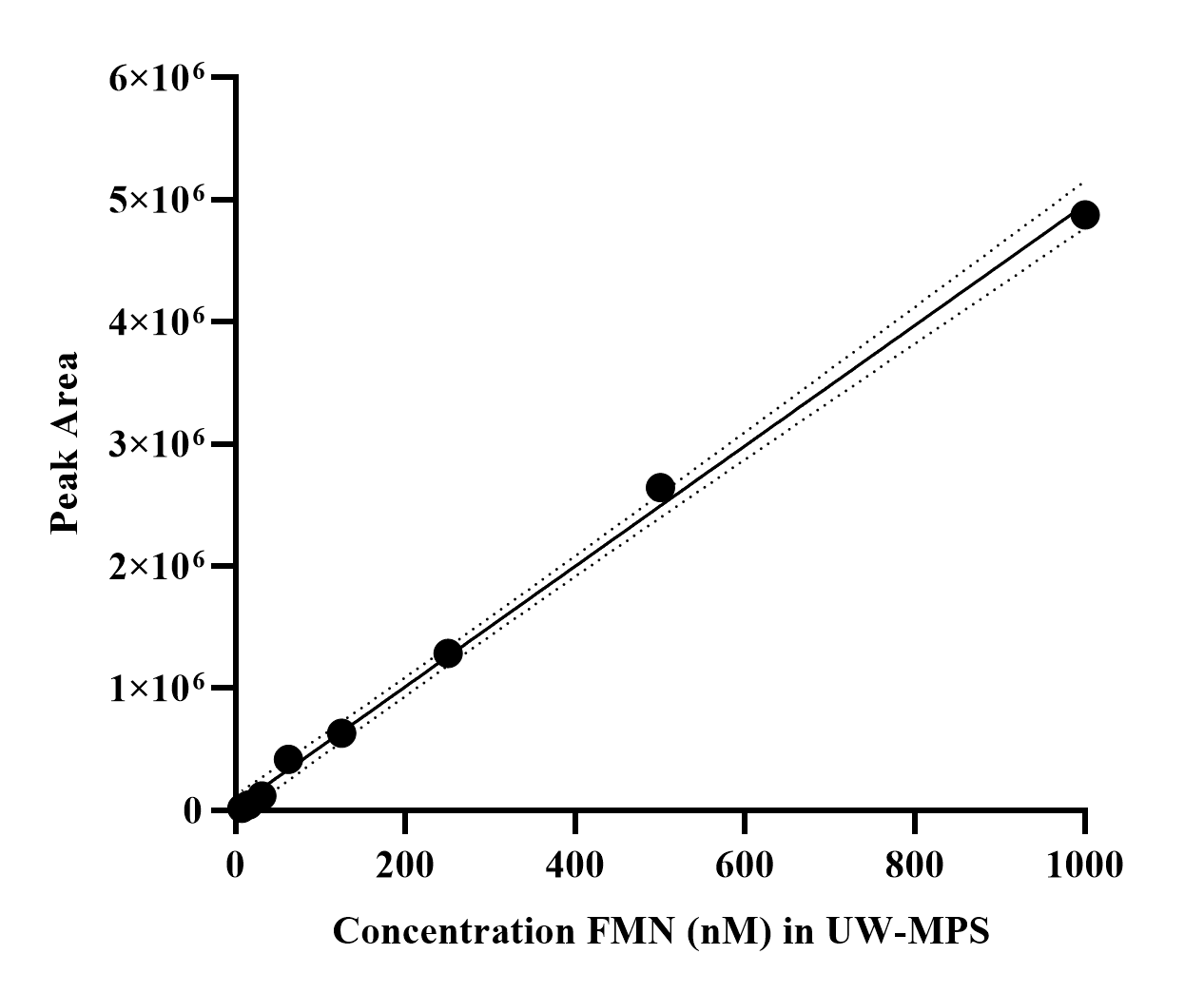

Supplement: S3 Fig — An eight point linear response was obtained after standard concentrations of FMN were diluted in perfusion fluid (UW-MPS) (Y = 4933.7×X+26465) (R2 = 0.9977). The limit of detection and limit of quantification of the LC-MS/MS assay were 0.05 picomoles. (TIF) [file pone.0287713.s003.tif]

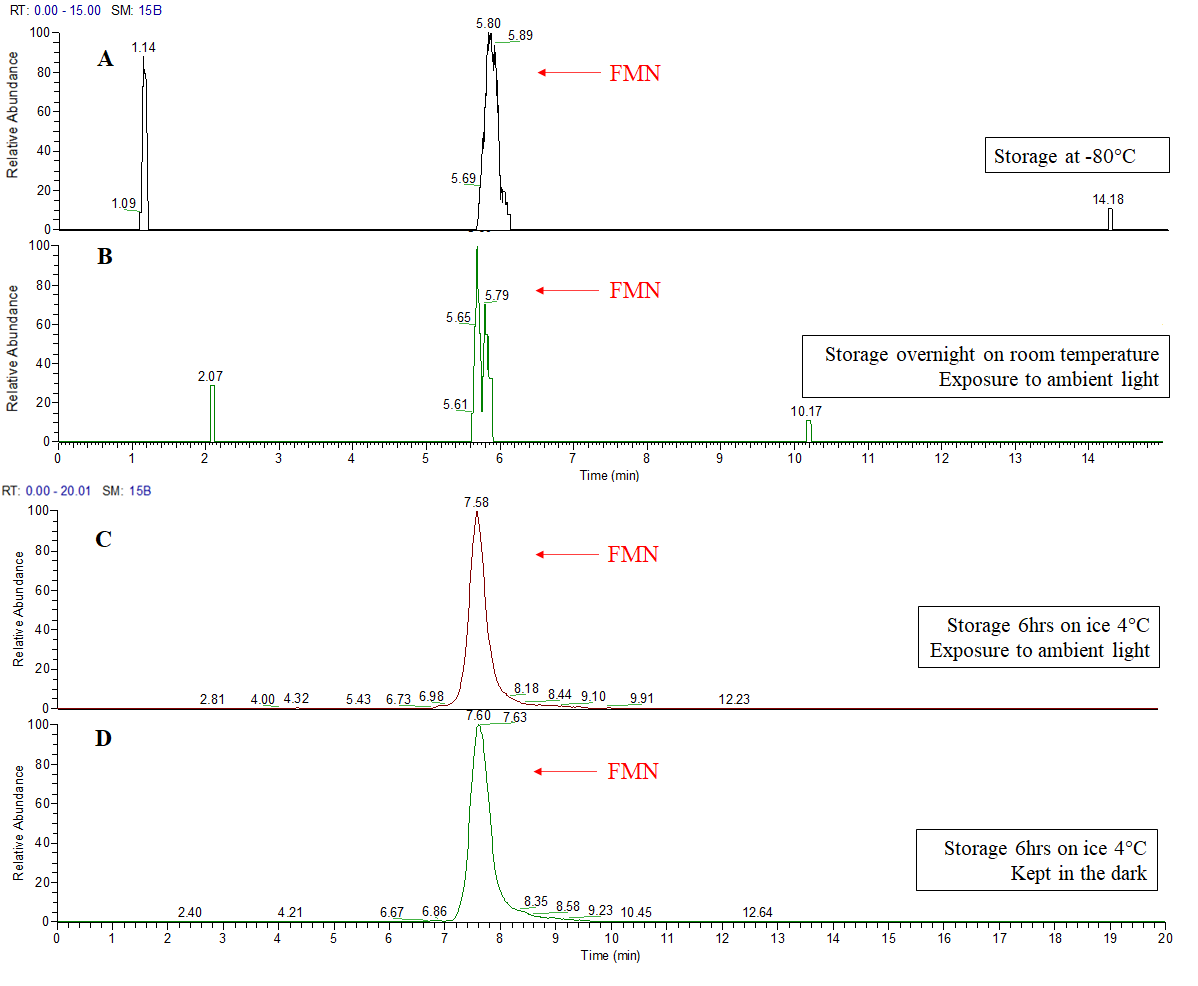

Supplement: S4 Fig — UW-MPS samples spiked with standard FMN concentration (500nM). (A) Storage at -80°C. (B) Storage overnight at room temperature and exposure to ambient light. (C) Storage for six hours on ice (4°C) and exposure to ambient light. (D) Storage for six hours on ice (4°C) and kept in the dark. (TIF) [file pone.0287713.s004.tif]
