# Supplementary material for: The role of flavin mononucleotide (FMN) as a potentially clinically relevant biomarker to predict the quality of kidney grafts during hypothermic (oxygenated) machine perfusion
Source: PLoS One. 2023 Jun 23;18(6):e0287713. doi: 10.1371/journal.pone.0287713 (PMC10289320; doi:10.1371/journal.pone.0287713)
Supplement: S1 Table — Serum creatinine was measured in patients with an immediate functioning graft not requiring dialysis treatment in the first week after transplantation. This was then correlated with fluorescence intensity (FI)(ex450;em500-600) measured at the beginning of perfusion (P1), during perfusion (P2) and the delta perfusion (ΔP = P3-P1). Spearman correlation test was used. (DOCX) [file pone.0287713.s005.docx]

| **Table S1. Correlation of Fluorescence Intensity (FI)_(ex450;em500-600)_** **at the beginning of perfusion (P1), during perfusion (P2) and the delta perfusion (∆P) with early post transplantation outcomes.** | | | | | | |  |
| --- | --- | --- | --- | --- | --- | --- | --- |
|  | **P1** | | **P2** | | **∆P** | |  |
|  | **R** | **p-value** | **R** | **p-value** | **R** | **p-value** | |
| Serum creatinine (µmol/L)  Day 5 | 0.255 | 0.06 | 0.292 | 0.03 | -0.181 | 0.19 | |
| Serum creatinine (µmol/L)  Day 7 | 0.238 | 0.08 | 0.334 | 0.01 | -0.135 | 0.33 | |
| Serum creatinine was measured in patients with an immediate functioning graft not requiring dialysis treatment in the first week after transplantation. This was then correlated with fluorescence intensity (FI)_(ex450;em500-600)_ measured at the beginning of perfusion (P1) during perfusion (P2) and the delta perfusion (∆P = P3-P1). Spearman correlation test was used.  FI, fluorescence intensity. | | | | | | |  |
