# Supplementary material for: The role of flavin mononucleotide (FMN) as a potentially clinically relevant biomarker to predict the quality of kidney grafts during hypothermic (oxygenated) machine perfusion
Source: PLoS One. 2023 Jun 23;18(6):e0287713. doi: 10.1371/journal.pone.0287713 (PMC10289320; doi:10.1371/journal.pone.0287713)
Supplement: S2 Table — † Pearson correlation test was used for correlation between creatinine clearance and FI(ex450;em500-600) measured in perfusates taken at the beginning of perfusion (P1). ‡ Logistic regression analyses were used for association between FI(ex450;em500-600) and graft failure or rejection. With regards to rejection at one year, the numbers were too small to perform the analysis. Data are presented as correlation coefficient (r) or odds ratio with corresponding [95% Confidence Interval]. (DOCX) [file pone.0287713.s006.docx]

| **Table S2. Association of Fluorescence Intensity (FI)_(ex450;em500-600)_ at the beginning of perfusion (P1) with post transplantation outcomes.** | | | | | | |
| --- | --- | --- | --- | --- | --- | --- |
|  | **3 months** | | **6 months** | | **1 year** | |
|  |  | **p-value** |  | **p-value** |  | **p-value** |
| Creatinine clearance ^†^ | -0.145 | 0.18 | -0.016 | 0.89 | -0.139 | 0.21 |
| Graft failure ^‡^ | 0.981 [0.96-1.00] | 0.09 | 0.981 [0.96-1.00] | 0.09 | 0.985 [0.97-1.00] | 0.15 |
| Rejection ^‡^ | 0.993 [0.98-1.00] | 0.21 | 0.995 [0.98-1.01] | 0.52 | X | X |
| ^†^ Pearson correlation test was used for correlation between creatinine clearance and FI_(ex450;em500-600)_ measured in perfusates taken at the beginning of perfusion (P1).  ^‡^ Logistic regression analyses were used for association between FI_(ex450;em500-600)_ and graft failure or rejection. With regards to rejection at one year, the numbers were too small to perform the analysis.  Data are presented as correlation coefficient (r) or odds ratio (OR) with corresponding [95% CI].  CI, Confidence Interval; FI, fluorescence intensity; OR, odds ratio. | | | | | | |
