# Supplementary material for: The role of flavin mononucleotide (FMN) as a potentially clinically relevant biomarker to predict the quality of kidney grafts during hypothermic (oxygenated) machine perfusion
Source: PLoS One. 2023 Jun 23;18(6):e0287713. doi: 10.1371/journal.pone.0287713 (PMC10289320; doi:10.1371/journal.pone.0287713)
Supplement: S1 Appendix — FMN analysis in perfusate using targeted liquid chromatography mass spectrometry. (DOCX) [file pone.0287713.s009.docx]

**S1 Appendix. Material & Methods**

FMN analysis in perfusate using targeted liquid chromatography mass spectrometry.

An aliquot of each perfusate sample (100µL) and serial FMN dilutions (ranging from 10nM to 1000nM), were mixed with HPLC-grade water, methanol and chloroform (1:1:2) and subsequently centrifuged (13.000 RPM, 20 minutes at 4°C). The upper phase, containing polar metabolites, was collected, lyophilized and stored at -20°C. Lyophilized metabolite fractions were individually reconstituted in 20µL of loading mobile phase with solvent A (2% Acetonitrile, 0.1% Formic Acid (FA)), and 10µL was loaded by a Dionex Ultimate 3000 (Thermo Scientific) at 200µL/minute on a Luna C^18^ column (2mm ID x 10cm length, 5µm particle size, Phenomenex Inc., Macclesfield, UK). A linear gradient of 3 to 90 % solvent B (100% Acetonitrile, 0.1% FA), was applied for 11 minutes for the separation of the target metabolites, followed by a column wash for 3 minutes (solvent B) and 6 minutes equilibration with solvent A. The total run time was 20 minutes.

LC-MS/MS analysis of eluting metabolites was conducted through Xcalibur 4.0 with Foundation 3.1 SP1 on an Orbitrap Fusion mass spectrometer (Thermo Scientific) in positive polarity mode, coupled to heated electrospray ionisation at 3.5 kV, Sheath Gas: 35, Aux Gas: 10, Ion Transfer Tube Temp: 300°C and Vaporizer Temp: 300°C. All MS1 acquisitions were performed between scan range 100-1000 (m/z) at 60K Orbitrap Resolution, with 100ms maximum injection time. MS2 of all target metabolites was acquired between mass range (m/z) 50-700, using fixed Higher-energy Collisional Dissociation of 40.

The FMN precursor (m/z~457.11) and four dominant fragments (m/z~172.09, 243.08, 359.13, 439.10) were used for identification and quantification of the analyte in standards and perfusate samples.

For modified products of FMN, “Find expected compounds” and “Predict composition” features were used in Compound Discoverer, with precursor mass tolerance of 5 part per million (ppm) and fragments mass tolerance of 5ppm, retention time (RT) tolerance of 6 seconds between samples. A blank sample (UW-MPS) was used to mark as background compounds and a mixture of spiked FMN standard and UW-MPS was used to define the modified products of FMN. KEGG and Human Metabolome database were used to identify metabolites.

In all LC-MS/MS experiments, three blanks were run between each sample and standards were analysed in order of increasing concentration to minimize sample carryover. LC-MS/MS conditions were optimised using standard FMN spiked into UW-MPS. Calibration curves were prepared in both positive and negative polarity mode on Orbitrap Fusion, before quantitative analysis of perfusate samples.
