# Supplementary material for: The role of flavin mononucleotide (FMN) as a potentially clinically relevant biomarker to predict the quality of kidney grafts during hypothermic (oxygenated) machine perfusion
Source: PLoS One. 2023 Jun 23;18(6):e0287713. doi: 10.1371/journal.pone.0287713 (PMC10289320; doi:10.1371/journal.pone.0287713)
Supplement: S2 Appendix — (DOCX) [file pone.0287713.s010.docx]

**S2 Appendix. Protocol fluorescence measurements of FMN 21/01/2020**

**Aim:** FMN levels in HMP and HMPO_2_ perfusates will be analysed by fluorescence using a microplate reader (BMG, CLARIOstar). Negative control (UW-MPS); FMN standards (serially diluted) and samples will be analysed following the protocol and plate layout below.

**Materials:**

- UW-MPS (Bridge to life)
- FMN (F2253, Sigma Aldrich) MW 478.33 g/mol
- MilliQ H_2_O
- 96-well black plates, with clear bottom

**Protocol:**

- Weigh 0.0143g (14.3 mg) of FMN and dissolve them in 3mL milliQ H_2_O to obtain a 10mM stock solution
- Dilute stock FMN solution 1:100 in UW-MPS to obtain 1mL FMN 100uM
- Dilute 39uL FMN 100uM in 5mL UW-MPS to obtain FMN 0.78uM (780nM) (highest std)
- Serially dilute stds 1:5 in UW-MPS solution (i.e. 400uL FMN + 1600uL UW-MPS)
- Plate 150uL/well of negative control, stds and samples according to plate layout below. (Assess samples and stds in duplicate)
- Read plate using the following parameters

Fluorescence Intensity in previously described FMN region: excitation 450nm, emission 500-600nm

|  | **1** | **2** | **3** | **4** | **5** | **6** | **7** | **8** | **9** | **10** | **11** | **12** |
| --- | --- | --- | --- | --- | --- | --- | --- | --- | --- | --- | --- | --- |
| **A** | FMN 780nM | | UW-MPS | |  | |  | |  | |  | |
| **B** | FMN 156nM | |  | |  | |  | |  | |  | |
| **C** | FMN 31.2nM | |  | |  | |  | |  | |  | |
| **D** | FMN 6.24nM | |  | |  | |  | |  | |  | |
| **E** | FMN 1.25nM | |  | |  | |  | |  | |  | |
| **F** | FMN 0.25nM | |  | |  | |  | |  | |  | |
| **G** | FMN 0.05nM | |  | |  | |  | |  | |  | |
| **H** | FMN 0.01nM | |  | |  | |  | |  | |  | |

**Plate layout**

(Run 39 samples/plate in duplicate).
