# Supplementary material for: The role of flavin mononucleotide (FMN) as a potentially clinically relevant biomarker to predict the quality of kidney grafts during hypothermic (oxygenated) machine perfusion
Source: PLoS One. 2023 Jun 23;18(6):e0287713. doi: 10.1371/journal.pone.0287713 (PMC10289320; doi:10.1371/journal.pone.0287713)
Supplement: S1 File — (DOCX) [file pone.0287713.s013.docx]

**Supplemental Material**

**The role of flavin mononucleotide (FMN) as a potentially clinically relevant biomarker to predict the quality of kidney grafts during hypothermic (oxygenated) machine perfusion.**

Fenna E.M. van de Leemkolk, M. Letizia Lo Faro, Sadr Shaheed, John F. Mulvey, Volkert A.L. Huurman, Ian P.J. Alwayn, Hein Putter, Ina Jochmans, Jan H.N. Lindeman, Rutger J. Ploeg.

On behalf of the COMPARE Trial Collaboration and Consortium for Organ Preservation in Europe (COPE)

**Table of Content**

**Title and authors 1**

**Table of Content 2**

**Material & Methods 3**

**Results 4**

**Supporting information captions 5-6**

**Supplemental Figures 7-10**

**Supplemental Tables 11-14**

**Protocol fluorescence measurements of FMN 15**

**Material & Methods**

FMN analysis in perfusate using targeted liquid chromatography mass spectrometry.

An aliquot of each perfusate sample (100µL) and serial FMN dilutions (ranging from 10nM to 1000nM), were mixed with HPLC-grade water, methanol and chloroform (1:1:2) and subsequently centrifuged (13.000 RPM, 20 minutes at 4°C). The upper phase, containing polar metabolites, was collected, lyophilized and stored at -20°C. Lyophilized metabolite fractions were individually reconstituted in 20µL of loading mobile phase with solvent A (2% Acetonitrile, 0.1% Formic Acid (FA)), and 10µL was loaded by a Dionex Ultimate 3000 (Thermo Scientific) at 200µL/minute on a Luna C^18^ column (2mm ID x 10cm length, 5µm particle size, Phenomenex Inc., Macclesfield, UK). A linear gradient of 3 to 90 % solvent B (100% Acetonitrile, 0.1% FA), was applied for 11 minutes for the separation of the target metabolites, followed by a column wash for 3 minutes (solvent B) and 6 minutes equilibration with solvent A. The total run time was 20 minutes.

LC-MS/MS analysis of eluting metabolites was conducted through Xcalibur 4.0 with Foundation 3.1 SP1 on an Orbitrap Fusion mass spectrometer (Thermo Scientific) in positive polarity mode, coupled to heated electrospray ionisation at 3.5 kV, Sheath Gas: 35, Aux Gas: 10, Ion Transfer Tube Temp: 300°C and Vaporizer Temp: 300°C. All MS1 acquisitions were performed between scan range 100-1000 (m/z) at 60K Orbitrap Resolution, with 100ms maximum injection time. MS2 of all target metabolites was acquired between mass range (m/z) 50-700, using fixed Higher-energy Collisional Dissociation of 40.

The FMN precursor (m/z~457.11) and four dominant fragments (m/z~172.09, 243.08, 359.13, 439.10) were used for identification and quantification of the analyte in standards and perfusate samples.

For modified products of FMN, “Find expected compounds” and “Predict composition” features were used in Compound Discoverer, with precursor mass tolerance of 5 part per million (ppm) and fragments mass tolerance of 5ppm, retention time (RT) tolerance of 6 seconds between samples. A blank sample (UW-MPS) was used to mark as background compounds and a mixture of spiked FMN standard and UW-MPS was used to define the modified products of FMN. KEGG and Human Metabolome database were used to identify metabolites.

In all LC-MS/MS experiments, three blanks were run between each sample and standards were analysed in order of increasing concentration to minimize sample carryover. LC-MS/MS conditions were optimised using standard FMN spiked into UW-MPS. Calibration curves were prepared in both positive and negative polarity mode on Orbitrap Fusion, before quantitative analysis of perfusate samples.

**Results**

Secondary endpoints.

No associations between Fluorescence Intensity(FI)_(ex450;em500-600)_ measured at the beginning of perfusion (P1), during perfusion (P2) or the delta perfusion (∆P = P3 – P1) and clinical post transplantation outcomes was evaluated (S1 Table, S2 Table, S3 Table and S4 Table). As a significant increase of FI_(ex450;em500-600)_ over collection time was observed (Figure 2, main manuscript) and a significant correlation was found between perfusion time (hrs) and FI_(ex450;em500-600)_ for P3, the timepoints P1 and P2 are less clinically relevant. P<0.05 was considered significant (p<0.0045 after Bonferroni correction).

**Supporting information captions:**

**S1 Fig. Fragmentation of FMN (m/z~457.11) on an Orbitrap Fusion mass spectrometer (ThermoScientific) in positive polarity mode.**
For optimal discrimination the FMN precursor (m/z~457.11) and four dominant fragments (m/z~172.087, 243.088, 359.136, 439.101) were used for identification and quantification of the analyte in standards and perfusate samples

**S2 Fig. Calibration curve fluorescence spectroscopy.**
A linear correlation was obtained after standard concentrations of FMN (nM) ranging from 6.2 to 780 nM were diluted in perfusion fluid (UW-MPS) $(Y=253.4\times X+953.7)$ (R^2^=0.9992; *p<0*.*0001*).

**S3 Fig. Calibration curve for targeted liquid chromatography mass spectrometry.**
An eight point linear response was obtained after standard concentrations of FMN were diluted in perfusion fluid (UW-MPS) $(Y=4933.7\times X+26465)$ (R2=0.9977). The limit of detection and limit of quantification of the LC-MS/MS assay were 0.05 picomoles.

**S4 Fig. Representative LC-MS/MS chromatograms of FMN (m/z 457.11) during stability experiments.**
UW-MPS samples spiked with standard FMN concentration (500nM). (A) Storage at -80°C. (B) Storage overnight at room temperature and exposure to ambient light. (C) Storage for six hours on ice (4°C) and exposure to ambient light. (D) Storage for six hours on ice (4°C) and kept in the dark..

**S1 Table. Correlation of Fluorescence Intensity (FI)_(ex450;em500-600)_** **at the beginning of perfusion (P1), during perfusion (P2) and the delta perfusion (∆P) with early post transplantation outcomes.**
Serum creatinine was measured in patients with an immediate functioning graft not requiring dialysis treatment in the first week after transplantation. This was then correlated with fluorescence intensity (FI)_(ex450;em500-600)_ measured at the beginning of perfusion (P1), during perfusion (P2) and the delta perfusion (∆P = P3-P1). Spearman correlation test was used.

**S2 Table.** **Association of Fluorescence Intensity (FI)_(ex450;em500-600)_ at the beginning of perfusion (P1) with post transplantation outcomes.**^†^ Pearson correlation test was used for correlation between creatinine clearance and FI_(ex450;em500-600)_ measured in perfusates taken at the beginning of perfusion (P1).
^‡^ Logistic regression analyses were used for association between FI_(ex450;em500-600)_ and graft failure or rejection. With regards to rejection at one year, the numbers were too small to perform the analysis.
Data are presented as correlation coefficient (r) or odds ratio with corresponding [95% Confidence Interval].

**S3 Table. Association of Fluorescence Intensity (FI)_(ex450;em500-600)_ during perfusion (P2) with post transplantation outcomes.**^†^ Pearson correlation test was used for correlation between creatinine clearance and FI_(ex450;em500-600)_ measured in perfusates taken during perfusion (P2).
^‡^ Logistic regression analyses were used for association between FI_(ex450;em500-600)_ and graft failure or rejection. With regards to rejection at one year, the numbers were too small to perform the analysis.
Data are presented as correlation coefficient (r) or odds ratio with corresponding [95% Confidence Interval].

**S4 Table. Association of Fluorescence Intensity (FI)_(ex450;em500-600)_ from the delta perfusion (∆P) with post transplantation outcomes.**^†^ Spearman correlation test was used for correlation between creatinine clearance and FI_(ex450;em500-600)_ from the delta perfusion (∆P) measured as (P3) perfusates taken at the end of perfusion – (P1) perfusates taken at the beginning of perfusion.
^‡^ Logistic regression analyses were used for association between FI_(ex450;em500-600)_ and graft failure or rejection. With regards to rejection at one year, the numbers were too small to perform the analysis.
Data are presented as correlation coefficient (r) or odds ratio with corresponding [95% Confidence Interval].

**Supplemental Figure 1**


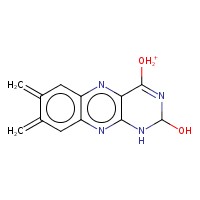

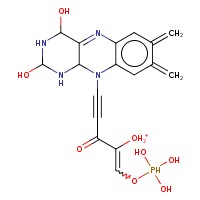

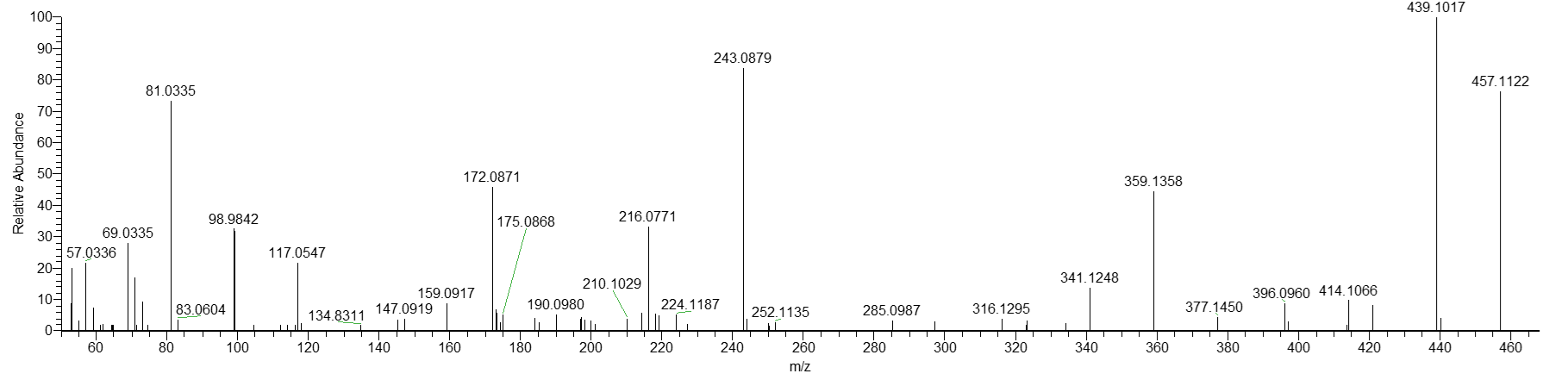

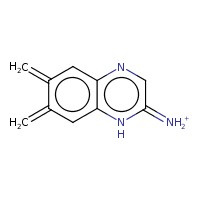

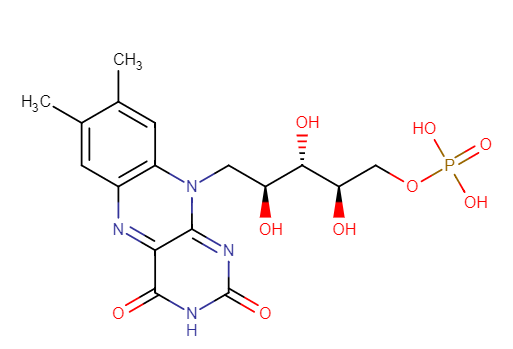

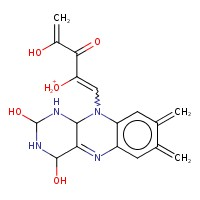


**FMN**

**
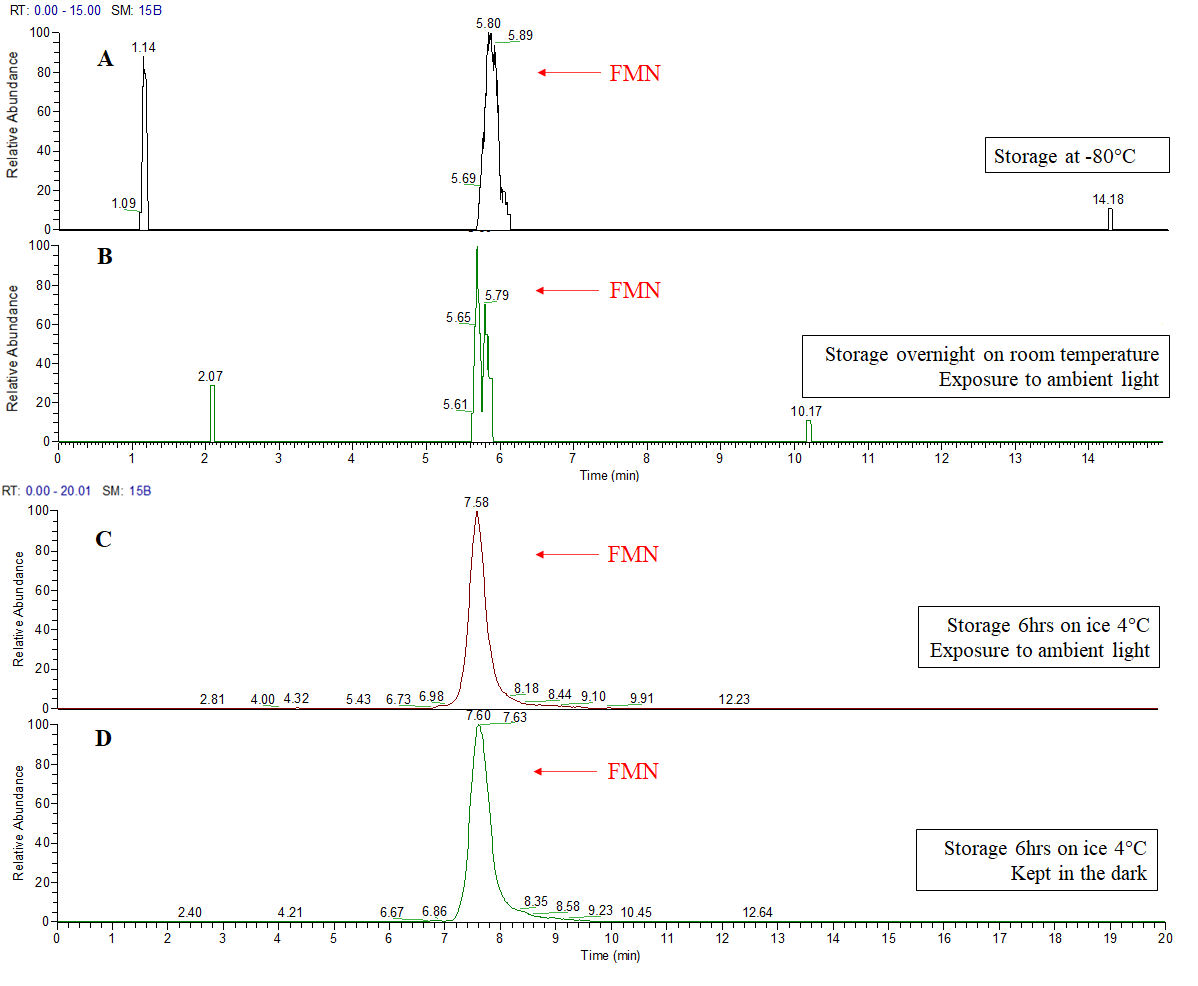
**

**Supplemental Figure 4**

| **Table S1. Correlation of Fluorescence Intensity (FI)_(ex450;em500-600)_** **at the beginning of perfusion (P1), during perfusion (P2) and the delta perfusion (∆P) with early post transplantation outcomes.** | | | | | | |  |
| --- | --- | --- | --- | --- | --- | --- | --- |
|  | **P1** | | **P2** | | **∆P** | |  |
|  | **R** | **p-value** | **R** | **p-value** | **R** | **p-value** | |
| Serum creatinine (µmol/L)  Day 5 | 0.255 | 0.06 | 0.292 | 0.03 | -0.181 | 0.19 | |
| Serum creatinine (µmol/L)  Day 7 | 0.238 | 0.08 | 0.334 | 0.01 | -0.135 | 0.33 | |
| Serum creatinine was measured in patients with an immediate functioning graft not requiring dialysis treatment in the first week after transplantation. This was then correlated with fluorescence intensity (FI)_(ex450;em500-600)_ measured at the beginning of perfusion (P1) during perfusion (P2) and the delta perfusion (∆P = P3-P1). Spearman correlation test was used.  FI, fluorescence intensity. | | | | | | |  |

| **Table S2. Association of Fluorescence Intensity (FI)_(ex450;em500-600)_ at the beginning of perfusion (P1) with post transplantation outcomes.** | | | | | | |
| --- | --- | --- | --- | --- | --- | --- |
|  | **3 months** | | **6 months** | | **1 year** | |
|  |  | **p-value** |  | **p-value** |  | **p-value** |
| Creatinine clearance ^†^ | -0.145 | 0.18 | -0.016 | 0.89 | -0.139 | 0.21 |
| Graft failure ^‡^ | 0.981 [0.96-1.00] | 0.09 | 0.981 [0.96-1.00] | 0.09 | 0.985 [0.97-1.00] | 0.15 |
| Rejection ^‡^ | 0.993 [0.98-1.00] | 0.21 | 0.995 [0.98-1.01] | 0.52 | X | X |
| ^†^ Pearson correlation test was used for correlation between creatinine clearance and FI_(ex450;em500-600)_ measured in perfusates taken at the beginning of perfusion (P1).  ^‡^ Logistic regression analyses were used for association between FI_(ex450;em500-600)_ and graft failure or rejection. With regards to rejection at one year, the numbers were too small to perform the analysis.  Data are presented as correlation coefficient (r) or odds ratio (OR) with corresponding [95% CI].  CI, Confidence Interval; FI, fluorescence intensity; OR, odds ratio. | | | | | | |

| **Table S3. Association of Fluorescence Intensity (FI)_(ex450;em500-600)_ during perfusion (P2) with post transplantation outcomes** | | | | | | |
| --- | --- | --- | --- | --- | --- | --- |
|  | **3 months** | | **6 months** | | **1 year** | |
|  |  | **p-value** |  | **p-value** |  | **p-value** |
| Creatinine clearance ^†^ | -0.242 | 0.03 | -0.028 | 0.81 | -0.214 | 0.06 |
| Graft failure ^‡^ | 0.994  [098-1.01] | 0.52 | 0.992  [0.97-1.01] | 0.39 | 0.993  [0.97-1.01] | 0.45 |
| Rejection ^‡^ | 0.994  [0.98-1.00] | 0.33 | 1.00  [0.98-1.02] | 0.97 | X | X |
| ^†^ Pearson correlation test was used for correlation between creatinine clearance and FI_(ex450;em500-600)_ measured in perfusates taken during perfusion (P2).  ^‡^ Logistic regression analyses were used for association between FI_(ex450;em500-600)_ and graft failure or rejection. With regards to rejection at one year, the numbers were too small to perform the analysis. Data are presented as correlation coefficient (r) or odds ratio (OR) with corresponding [95% CI].  CI, Confidence Interval; FI, fluorescence intensity; OR, odds ratio. | | | | | | |

| **Table S4. Association of Fluorescence Intensity (FI)_(ex450;em500-600)_ from the delta perfusion (∆P) with post transplantation outcomes.** | | | | | | |
| --- | --- | --- | --- | --- | --- | --- |
|  | **3 months** | | **6 months** | | **1 year** | |
|  |  | **p-value** |  | **p-value** |  | **p-value** |
| Creatinine clearance ^†^ | 0.05 | 0.66 | 0.06 | 0.61 | -0.118 | 0.30 |
| Graft failure ^‡^ | 1.00 [1.00-1.00] | 0.03 | 1.00 [1.00-1.00] | 0.06 | 1.00 [1.00-1.00] | 0.07 |
| Rejection ^‡^ | 1.00 [1.00-1.00] | 0.67 | 1.00 [1.00-1.00] | 0.78 | X | X |
| ^†^ Spearman correlation test was used for correlation between creatinine clearance and FI_(ex450;em500-600)_ from the delta perfusion (∆P) measured as (P3) perfusates taken at the end of perfusion – (P1) perfusates taken at the beginning of perfusion.  ^‡^ Logistic regression analyses were used for association between FI_(ex450;em500-600)_ and graft failure or rejection. With regards to rejection at one year, the numbers were too small to perform the analysis.  Data are presented as correlation coefficient (r) or odds ratio (OR) with corresponding [95% CI].  CI, Confidence Interval; FI, fluorescence intensity; OR, odds ratio. | | | | | | |

**Protocol fluorescence measurements of FMN 21/01/2020**

**Aim:** FMN levels in HMP and HMPO_2_ perfusates will be analysed by fluorescence using a microplate reader (BMG, CLARIOstar). Negative control (UW-MPS); FMN standards (serially diluted) and samples will be analysed following the protocol and plate layout below.

**Materials:**

- UW-MPS (Bridge to life)
- FMN (F2253, Sigma Aldrich) MW 478.33 g/mol
- MilliQ H_2_O
- 96-well black plates, with clear bottom

**Protocol:**

- Weigh 0.0143g (14.3 mg) of FMN and dissolve them in 3mL milliQ H_2_O to obtain a 10mM stock solution
- Dilute stock FMN solution 1:100 in UW-MPS to obtain 1mL FMN 100uM
- Dilute 39uL FMN 100uM in 5mL UW-MPS to obtain FMN 0.78uM (780nM) (highest std)
- Serially dilute stds 1:5 in UW-MPS solution (i.e. 400uL FMN + 1600uL UW-MPS)
- Plate 150uL/well of negative control, stds and samples according to plate layout below. (Assess samples and stds in duplicate)
- Read plate using the following parameters

Fluorescence Intensity in previously described FMN region: excitation 450nm, emission 500-600nm

|  | **1** | **2** | **3** | **4** | **5** | **6** | **7** | **8** | **9** | **10** | **11** | **12** |
| --- | --- | --- | --- | --- | --- | --- | --- | --- | --- | --- | --- | --- |
| **A** | FMN 780nM | | UW-MPS | |  | |  | |  | |  | |
| **B** | FMN 156nM | |  | |  | |  | |  | |  | |
| **C** | FMN 31.2nM | |  | |  | |  | |  | |  | |
| **D** | FMN 6.24nM | |  | |  | |  | |  | |  | |
| **E** | FMN 1.25nM | |  | |  | |  | |  | |  | |
| **F** | FMN 0.25nM | |  | |  | |  | |  | |  | |
| **G** | FMN 0.05nM | |  | |  | |  | |  | |  | |
| **H** | FMN 0.01nM | |  | |  | |  | |  | |  | |

**Plate layout**

(Run 39 samples/plate in duplicate).
